# 

**Published:** 1891-04-04

**Authors:** 


FOR INFANTS AND INVALIDS.

### THE HOSPITAL

WITH EXTRA NURSING SUPPLEMENT.

No. 236. Vol. X.] SATURDAY, APRIL 4th, 1891. [ONE PENNY.

